# Successful interval laparoscopic appendectomy for walled-off perforated appendicitis during pregnancy: a case report

**DOI:** 10.1097/RC9.0000000000000911

**Published:** 2026-09-02

**Authors:** Shinichiro Usuki, Kana Murakami, Anju Kawaguchi

**Affiliations:** a Department of General Surgery, Tokyo-Kita Medical Center, Tokyo, Japan; b Department of Obstetrics and Gynecology, Tokyo-Kita Medical Center, Tokyo, Japan

**Keywords:** appendicitis in pregnancy, case report, interval laparoscopic appendectomy, walled-off perforated appendicitis

## Abstract

**Introduction:**

Acute appendicitis is the most common non-obstetric surgical emergency during pregnancy, with an incidence of approximately 0.067% (1 in 1493 pregnancies). Although prompt laparoscopic appendectomy is the standard treatment, optimal management of walled-off perforated appendicitis (WOPA) during pregnancy is unclear.

**Case presentation:**

A 28-year-old woman presented with a 1-week history of abdominal pain and fever at 9 weeks of gestation. Ultrasonography demonstrated an ill-defined right lower quadrant mass, and contrast-enhanced computed tomography revealed WOPA with a localized abscess clearly separated from the uterus. Following a multidisciplinary discussion with the obstetric team, initial nonoperative management (NOM) with intravenous antibiotics was considered because the patient was clinically stable and the inflammation was localized. The patient’s symptoms resolved without complications, and she was discharged after 11 days. Elective laparoscopic interval appendectomy (IA) was successfully performed 9 weeks later, at 18 weeks of gestation during the second trimester. Only mild adhesions were observed intraoperatively, and the postoperative course was uneventful. The pregnancy progressed without any maternal or fetal complications.

**Discussion:**

In this case, delayed presentation, localized abscess formation, clinical stability, and anatomical separation of the inflammatory lesion from the uterus supported the initial conservative approach. Elective IA performed during the second trimester avoided technically demanding emergency surgery and was safely completed. This case, together with the limited published literature, suggests that staged management may be appropriate among carefully selected pregnant women.

**Conclusion:**

In carefully selected pregnant patients with WOPA, initial NOM followed by interval laparoscopic appendectomy may be safe and feasible. Additional case studies are required to establish the optimal management recommendations for this rare clinical condition.

## Introduction

Acute appendicitis during pregnancy is the most common non-obstetric surgical emergency, with an incidence of approximately 0.067%[1]. Prompt appendectomy is generally recommended, and laparoscopic appendectomy is safe in pregnant patients[2]. Free perforations require emergency surgery owing to the increased risk of preterm labor and fetal loss[3]. However, the optimal management of walled-off perforated appendicitis (WOPA), particularly during pregnancy, remains unclear. We report the successful management of WOPA in a 28-year-old woman with initial nonoperative management (NOM) followed by interval laparoscopic appendectomy. This case report is documented in accordance with the SCARE 2025 criteria[4].

HIGHLIGHTS

- Walled-off perforated appendicitis during pregnancy is a rare clinical entity with no established treatment.
- Initial nonoperative management successfully controlled localized infections and avoided the need for emergency surgery during early pregnancy.
- Interval laparoscopic appendectomy was safely performed during the second trimester, at 18 weeks of gestation, with favorable maternal and fetal outcomes.
- Careful patient selection based on clinical stability and localized disease may facilitate a staged treatment approach.
- This case provides evidence supporting the individualized, multidisciplinary management of pregnant patients with localized perforated appendicitis.

## Case presentation

### History

A 28-year-old woman at 9 weeks of gestation presented to the emergency department with a 1-week history of abdominal pain and fever. Four days before presentation, she was diagnosed with suspected gastroenteritis at a local clinic and treated with analgesics, but she showed no clinical improvement. She had no significant medical or surgical history. She was not taking any medications and had no known drug allergies.

### Examination

The patient was alert and hemodynamically stable. Her body temperature was 38.5°C, heart rate was 96 beats per minute, and respiratory rate was 24 breaths per minute. Abdominal examination revealed tenderness extending from the periumbilical region to the right lower quadrant.

### Lab investigation and imaging

Laboratory tests revealed leukocytosis (14280/mm) and elevated C-reactive protein (16.8 mg/dL). Ultrasonography revealed an ill-defined mass in the right lower quadrant; however, the appendix was not visible. After obtaining informed consent, contrast-enhanced computed tomography was performed, revealing a localized abscess consistent with WOPA and clearly separated from the uterus (Fig. 1).

**Figure 1.**
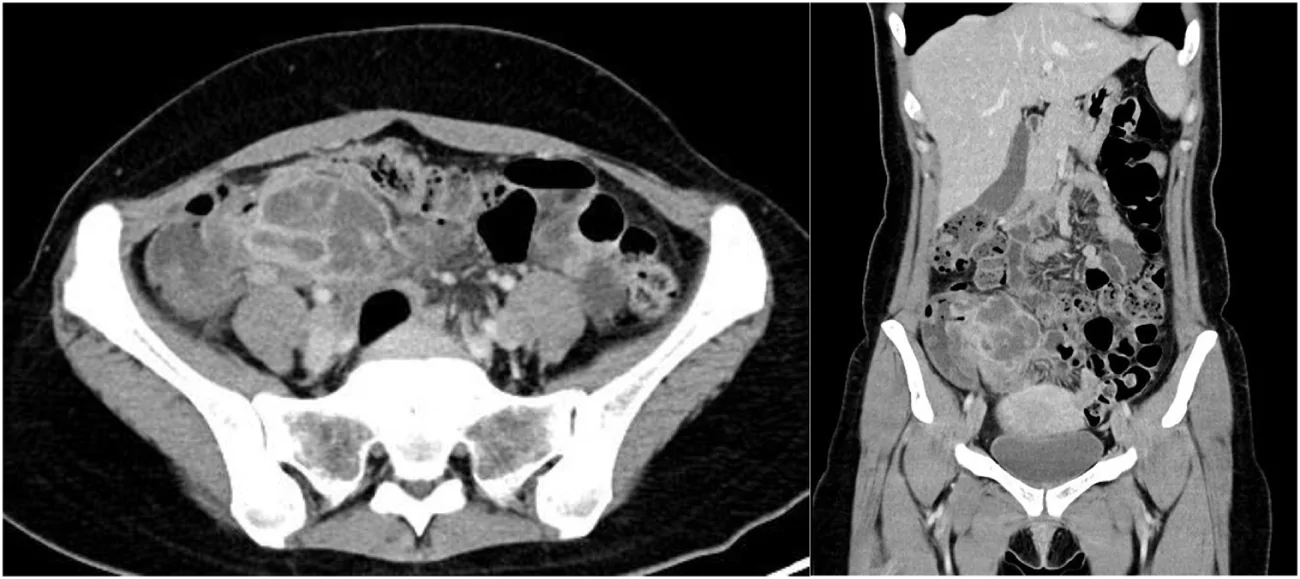
Contrast-enhanced computed tomography (axial and coronal views) shows a localized abscess, consistent with walled-off perforated appendicitis, that is clearly separated from the uterus.

### Treatment and outcome

After a multidisciplinary discussion with the obstetric team, NOM with intravenous piperacillin–tazobactam was initiated. Her condition improved rapidly, with resolution of fever and gradual relief of abdominal pain. Oral intake was resumed on hospital day 3, and she was discharged on hospital day 11. Elective laparoscopic appendectomy was performed at 18 weeks of gestation, 9 weeks after the initial presentation. The procedure was performed by an experienced laparoscopic surgeon at a community-based hospital. The first port was placed at the umbilicus using an open (Hasson) technique, followed by two additional upper-abdominal ports. Intraoperatively, only mild adhesions were observed, and the appendix was safely resected (Fig. 2).

**Figure 2.**
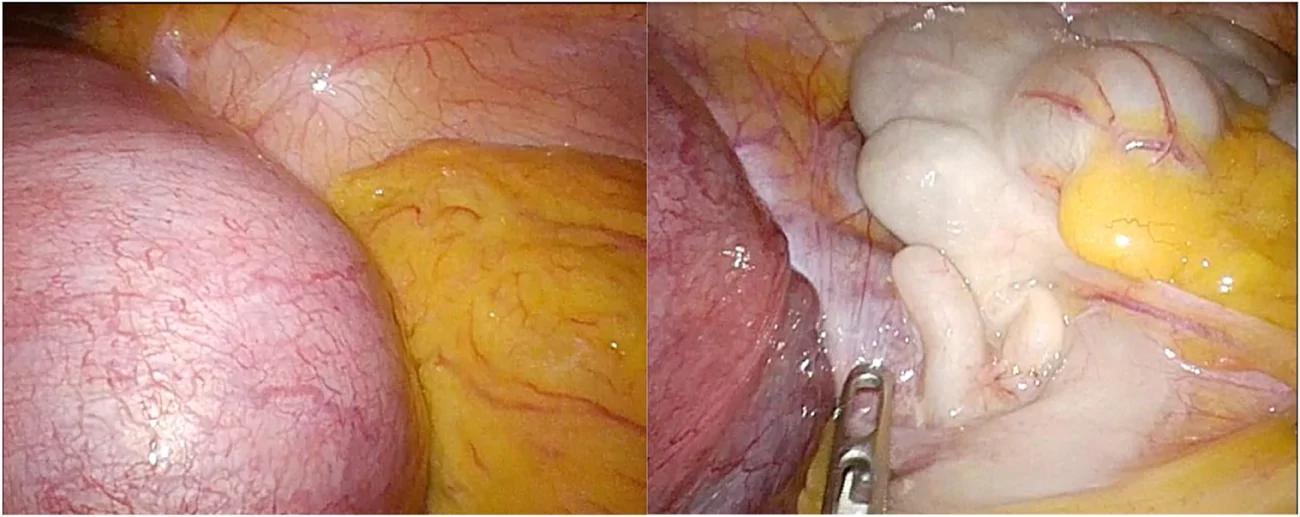
Intraoperative view showing an enlarged uterus and an appendix with only mild adhesions, located away from the uterus.

The postoperative course was uneventful, and she was discharged on postoperative day 3. The pregnancy remained uneventful. The clinical timeline is summarized in Table 1.

**Table 1:** Timeline of the clinical course.

Date	Clinical event
11 January 2026	Symptom onset
14 January 2026	Diagnosed with presumed gastroenteritis at a local clinic
18 January 2026	Presentation to the emergency department; contrast-enhanced computed tomography demonstrated walled-off perforated appendicitis
18–28 January 2026	Hospitalization and intravenous piperacillin–tazobactam
28 January 2026	Discharged after successful nonoperative management
23 March 2026	Elective interval laparoscopic appendectomy (18 weeks of gestation)

## Discussion

Appendicitis during pregnancy, a common non-obstetric surgical emergency, requires prompt management because it may lead to preterm labor and fetal loss, particularly in complicated cases[3]. Diagnosis is often challenging owing to the anatomical and physiological changes that occur during pregnancy. In this case, initial ultrasonographic imaging demonstrated only a vague mass in the right lower quadrant, and the appendix could not be visualized. Although ultrasonography is the first-line imaging modality, its diagnostic accuracy is limited in certain cases. Magnetic resonance imaging (MRI), a safe alternative during pregnancy[5], is not available at our hospital during nighttime emergencies. The estimated fetal radiation dose from a single abdominal CT scan is generally below the threshold for deterministic fetal effects (typically <50 mGy) and is therefore considered acceptable when clinically indicated during pregnancy[6], particularly because it facilitates accurate diagnosis and may prevent unnecessary surgical intervention. In this case, CT indicated WOPA, which was localized and anatomically separated from the uterus.

In non-pregnant patients with similar conditions, initial NOM with antibiotics is safe and feasible[7]. This strategy is generally favored because emergency surgery in the presence of severe inflammation and dense adhesions is associated with high morbidity, including the need for extended bowel resection and postoperative intra-abdominal abscess formation. In contrast, few reports have described the management of WOPA during pregnancy, and no consensus has been established. Young *et al* reported two cases of ruptured appendicitis during pregnancy managed successfully and nonoperatively with intravenous antibiotics. One patient had an uncomplicated vaginal delivery at term, followed by an interval laparoscopic appendectomy 2 months postpartum. The other patient experienced recurrence during pregnancy, required additional medical management, and ultimately underwent a cesarean delivery at 34 weeks of gestation, during which the appendix was removed[8]. Igarashi *et al* reported successful interval laparoscopic appendectomy at 17 weeks of gestation following initial abscess drainage and antibiotic therapy[9]. Degastani *et al* reported successful conservative management of phlegmonous appendicitis during pregnancy using intravenous antibiotics and close clinical follow-up without the need for surgical intervention[10]. These reports suggest that carefully selected pregnant patients with localized perforated appendicitis may be candidates for either conservative management alone or a staged approach consisting of initial NOM followed by interval appendectomy (IA).

In this case, several factors supported the decision to pursue NOM. First, the patient was clinically stable despite the perforation. Second, the duration since onset was relatively long, suggesting that the inflammatory process had localized. Third, CT revealed that the abscess was confined and located away from the uterus, indicating a limited effect on the fetus. Considering the potential need for extended bowel resection and the associated risks to both mother and fetus, the NOM strategy was appropriate.

After successful NOM, the optimal timing of IA remains unclear[11]. In non-pregnant patients, IA is generally performed 2–3 months after the initial treatment because the risk of recurrence is the highest within the first 6 months. The recurrence rate after NOM of perforated appendicitis with phlegmon or abscesses is 12% to 24%[5]. Youssef *et al* reported a recurrence rate of 17.6% (9/51), with 77.8% of the recurrences occurring within the first 6 months[12]. Although these data were derived from non-pregnant populations and recurrence rates during pregnancy remain unknown, recurrence may occur during an ongoing pregnancy and pose a greater clinical challenge owing to the enlarging uterus and increased technical difficulty of surgical intervention. Furthermore, IA is generally considered in patients aged over 40 years to exclude underlying appendiceal neoplasms[13]. However, because our patient was only 28 years old, the decision to perform IA was primarily based on the anticipated risk of recurrent appendicitis during the ongoing pregnancy, which outweighed the risks associated with elective IA. In this case, after multidisciplinary discussion with the obstetric team, interval laparoscopic appendectomy was scheduled for the second trimester, after 16 weeks of gestation, which is generally considered the preferred period for non-obstetric surgery during pregnancy. Accordingly, laparoscopic appendectomy was successfully performed at 18 weeks of gestation.

The principal strength of this study is the successful application of a staged treatment strategy comprising initial NOM followed by IA during pregnancy, resulting in favorable maternal and fetal outcomes. Appropriate patient selection based on clinical stability, multidisciplinary decision-making, and detailed radiological assessments contributed to successful outcomes. The limitations of this study include its single-case design, the lack of long-term maternal and neonatal outcome data, and the use of CT because MRI was unavailable in the emergency setting. Therefore, caution should be exercised when extrapolating these findings to other patients.

## Conclusion

Interval laparoscopic appendectomy following successful initial NOM can be safely performed during pregnancy in carefully selected patients. In the absence of a definitive treatment strategy, this approach may be safe and feasible in selected cases.

## Data Availability

All relevant data are included within the article. Further inquiries can be directed to the corresponding author.

## Artificial intelligence

No artificial intelligence was used in the preparation of this manuscript.
